# High-Altitude Cognitive Impairment Is Prevented by Enriched Environment Including Exercise via VEGF Signaling

**DOI:** 10.3389/fncel.2018.00532

**Published:** 2019-01-10

**Authors:** Christina Koester-Hegmann, Harkaitz Bengoetxea, Dmitry Kosenkov, Markus Thiersch, Thomas Haider, Max Gassmann, Edith M. Schneider Gasser

**Affiliations:** ^1^Neuroprotection Group, Institute of Pharmacology and Toxicology, University of Zurich, Zurich, Switzerland; ^2^Laboratory of Clinical and Experimental Neuroscience (LaNCE), Department of Neuroscience, Faculty of Medicine and Nursing, University of the Basque Country (UPV/EHU), Bilbao, Spain; ^3^Institute of Veterinary Physiology, Vetsuisse Faculty, University of Zurich, Zurich, Switzerland; ^4^Zurich Center for Integrative Human Physiology (ZIHP), University of Zurich, Zurich, Switzerland; ^5^Universidad Peruana Cayetano Heredia (UPCH), Lima, Peru; ^6^Center for Neuroscience Zurich (ZNZ), Zurich, Switzerland

**Keywords:** neuroprotection, neurogenesis, angiogenesis, tyrosine kinase inhibitor, spatial memory, visual memory

## Abstract

Exposure to hypobaric hypoxia at high altitude (above 2500 m asl) causes cognitive impairment, mostly attributed to changes in brain perfusion and consequently neuronal death. Enriched environment and voluntary exercise has been shown to improve cognitive function, to enhance brain microvasculature and neurogenesis, and to be neuroprotective. Here we show that high-altitude exposure (3540 m asl) of Long Evans rats during early adulthood (P48–P59) increases brain microvasculature and neurogenesis but impairs spatial and visual memory along with an increase in neuronal apoptosis. We tested whether enriched environment including a running wheel for voluntary exercise (EE) can prevent cognitive impairment at high-altitude and whether apoptosis is prevented. We found that EE retained spatial and visual memory at high altitude, and prevented neuronal apoptosis. Further, we tested whether vascular endothelial growth factor (VEGF) signaling is required for the EE-mediated recovery of spatial and visual memory and the reduction in apoptosis. Pharmacological inhibition of VEGF signaling by oral application of a tyrosine kinase inhibitor (Vandetanib) prevented the recovery of spatial and visual memory in animals housed in EE, along with an increase in apoptosis and a reduction in neurogenesis. Surprisingly, inhibition of VEGF signaling also caused impairment in spatial memory in EE-housed animals reared at low altitude, affecting mainly dentate gyrus microvasculature but not neurogenesis. We conclude that EE-mediated VEGF signaling is neuroprotective and essential for the maintenance of cognition and neurogenesis during high-altitude exposure, and for the maintenance of spatial memory at low altitude. Finally, our data also underlines the potential risk of cognitive impairment and disturbed high altitude adaption from the use of VEGF-signaling inhibitors for therapeutic purposes.

## Introduction

Exposure to hypobaric hypoxia after ascent to high altitude causes cognitive impairment, particularly in spatial and visuospatial information processing (Pagani et al., 1998; Virues-Ortega et al., 2004; Wilson et al., 2009; Nation et al., 2017), which involves the cornu ammonis (CA1) area of the hippocampal formation (Poucet et al., 2010) and the primary visual cortex (Prusky et al., 2004). One of the leading causes of memory dysfunction at high altitude is attributed to reductions in cerebral blood flow via the vascular network (Lawley et al., 2017), and consequently hypoxia, leading to apoptosis and neuronal loss (Titus et al., 2007; Maiti et al., 2008). Indeed, cerebral blood flow disturbances have been associated with decline in cognitive function, as well as with several types of dementia (Duncombe et al., 2017; Xu et al., 2017; Cubinkova et al., 2018; Kalaria, 2018). In a feedforward/feedback loop manner, the brain vascular network interacts with neurons as an organized functional unit, the neurovascular unit, in which cerebral vasculature can modulate neural activity and neural activity can dynamically adjust the cerebral blood flow (Moore and Cao, 2008).

Environmental enrichment and exercise (EE), both have shown to improve neural activity and consequently to enhance cerebral vasculature, and cognitive functions (Kempermann et al., 1997; Brown et al., 2003; Garthe et al., 2016; Ohline and Abraham, 2018). In hippocampus and cortex, EE can lead to secretion of neurotrophic factors such as brain-derived neurotrophic factor (Zoladz et al., 2008) and vascular endothelial growth factor (VEGF), the main hypoxia-inducible pro-angiogenic factor (Shweiki et al., 1992; Ding et al., 2006; Morland et al., 2017). In the central nervous system, VEGF is primarily expressed by astrocytes (Acker et al., 2001; Licht et al., 2011). During development, VEGF is also expressed in neural stem cells and progenitor cells, and is involved in neural stem cell maintenance and neurogenesis (Jin et al., 2002; Schanzer et al., 2004; Udo et al., 2008; Kirby et al., 2015; Licht et al., 2016). VEGF acts on vascular endothelial growth factor receptor-2 (VEGFR-2), expressed in vascular endothelial cells (Zachary and Gliki, 2001) and in neurons subjected to hypoxia, through the MAPK/ERK and PI3K/Akt signaling pathways (Fournier et al., 2012). VEGF has been shown to be neuroprotective *in vitro* by the enhanced survival of neurons in the presence of VEGF and, on the contrary, by increased apoptosis upon VEGF signaling blockade (Ogunshola et al., 2000). Both, exogenous VEGF administration and endogenous VEGF secretion were reported to restore ischemia-induced cognitive impairment *in vivo* and *in vitro* (Ortuzar et al., 2013; Yang et al., 2014). VEGF is also protective for vasculature in diseases such as vascular dementia (Park et al., 2017), Alzheimer’s disease (Religa et al., 2013), and post-focal traumatic brain injury (Ortuzar et al., 2013). Evidence for neuronal protection of VEGF was provided by studies showing that inhibition of VEGF signaling, via either monoclonal antibodies or tyrosine kinase inhibition, which inhibit vascular endothelial growth factor receptor-2 (VEGFR-2) (Noble et al., 2004), leads to impaired spatial memory and to a reduced number of neurons in rats (Pati et al., 2009; Bengoetxea et al., 2018).

Neurogenesis and improved cognition are usually interrelated and are both simulated by different physiological stimuli such as EE and hypoxia (Song et al., 2012; Varela-Nallar et al., 2014; Zhang et al., 2015). Adult neurogenesis in the subgranular layer (SGL) of the dentate gyrus occurs in close proximity to blood vessels (Palmer, 2002), giving rise to granular neurons and glia throughout the adult lifespan. Although a positive correlation between VEGF-induced hippocampal neurogenesis and cognition has been demonstrated previously (Ding et al., 2006; Varela-Nallar et al., 2014), the two are potentially not causally related, since blockade of VEGF signaling leads to impaired memory without reducing neurogenesis (Licht et al., 2011). Additionally, the effect of VEGF overexpression or inhibition on the gain/loss of memory is already measurable a few days after induction/blockade, a time window too short to consider neurogenesis as the factor responsible for improved memory (Foscarin et al., 2011, 2012). Further, hypoxia-induced neurogenesis is not sufficient to prevent cognitive impairment. The complex interplay between high altitude, enriched environment and VEGF signaling on angiogenesis, neurogenesis, neuroprotection and cognition is far from understood.

In the present study we hypothesized that exposure to EE in rats after rapid ascent to high altitude (3450 m asl) is neuroprotective and prevents spatial-visual memory impairment. Further, we hypothesize that EE-mediated VEGF signaling is required for the recovery of memory and neuroprotection, as well as for angiogenesis and neurogenesis. We therefore set out to identify, by the pharmacological inhibition of VEGF signaling, whether the effects of EE on neovasculature, neurogenesis, neuroprotection, and cognition differed between low and high altitude. We combined behavioral tests and morphological analysis of brain vasculature densities, cellular numbers and apoptotic neurons in the dentate gyrus, CA1 hippocampus and visual cortex.

## Materials and Methods

### Animals and Housing Conditions

All animal experiments were performed in accordance with the international guidelines on animal use and care and approved by the Animal Ethics Committee of the Cantonal Veterinary Office of Fribourg, Bern and Zürich (2011_32_FR).

**FIGURE 1:**
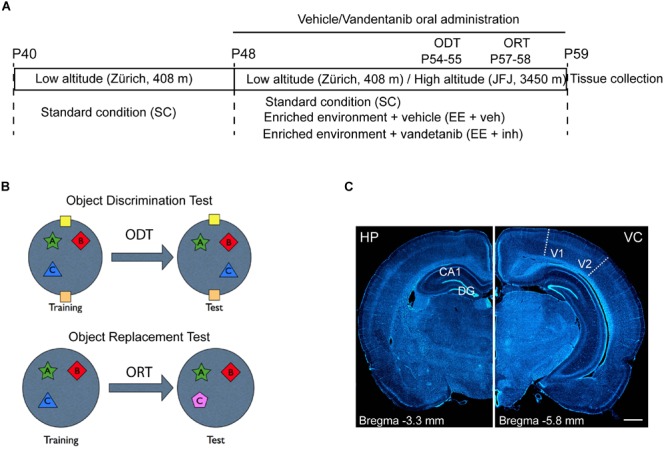
Experimental study design and brain areas of analysis. **(A)** Experimental study design. First housing: P40 to P48 at low altitude (Zürich, 408 m asl). Second housing: P48 to P59 either at low altitude (Zürich) or transported to the *High Altitude Research Station* Jungfraujoch (JFJ, 3450 m asl). ODT, Object Displacement Test (P54–P55). ORT, Object replacement test (P57–P58). Tissue collection: P59. Housing conditions: standard conditions (SC), enriched environment receiving sucrose (EE + veh), and enriched environment receiving Vandetanib (EE + inh). **(B)** ODT diagram for assessing spatial learning and ORT diagram for assessing visual learning tasks. **(C)** Representative images of coronal rat brain sections stained with DAPI showing in stereotaxic coordinates Bregma –3.3 mm the hippocampus (HP, left) and Bregma –5.8 mm the visual cortex (VC, right) and visualizing the areas used for morphological analysis: dentate gyrus (DG), and cornu ammonis 1 (CA1) in HP and visual area V1–V2 in VC. Scale bar: 1 mm.

Seventy-two Long Evans juvenile male rats (P40) were purchased from Janvier Labs (France). The experiments were conducted in two phases with 36 animals in each phase. The animals were randomly assigned to six different experimental conditions (*n* = 6 rats/group) as visualized schematically in Figure 1A. Rats were first housed from P40 to P48 in an animal facility at low altitude (Zürich, 408 m asl) in standard laboratory conditions (SC) at 22°C room temperature with a 12 h light/dark cycle and access to food and water *ad libitum*. At P48, half of the rats were kept in Zürich (low-altitude group), and the other half transported to the Jungfraujoch High Altitude Research Station on the Jungfraujoch (JFJ, 3450 m asl), (high-altitude group) in a single journey of 250 min duration. Both low- and high-altitude groups were then housed at 22°C room temperature with a 12 h light/dark cycle and access to food and water *ad libitum*, either in standard laboratory conditions (SC) or in an enriched environment that included voluntary exercise (EE) (Bengoetxea et al., 2013), and received either sucrose, (EE + veh), or the tyrosine kinase inhibitor Vandetanib (EE + inh).

Housing conditions:

Standard condition (SC): Cage size 630 mm L × 420 mm W × 220 mm H, with access to food and water *ad libitum*.

Enriched environment including voluntary exercise (EE): Cage size 800 mm L × 490 mm W × 630 mm H (Cometa Rodent, Imac, Italy). The cage included different shaped objects, bridges and tunnels for play, and one running wheel as a voluntary exercise option (Wodent Wheels Wobust 30 cm), with food and water in different places *ad libitum*.

### Vandetanib Administration

Vandetanib (C_22_H_24_BrFN_4_O_2_), also known as ZD6474, is an active tyrosine kinase inhibitor which selectively inhibits VEGFR2-dependent angiogenesis (Herbst et al., 2007). Vandetanib was purchased from LC Laboratories, United States (Ref. V9402 Vandetanib, United States) and orally applied daily (30 mg/kg/day). The drug amount was calculated according to the actual body weight of treated rats (EE + inh) and dissolved with the help of a sonicator in saline solution (NaCl 0.9%) that included 1% Tween 80 (Ref. P1754 Sigma-Aldrich) and 5% sucrose (saccharose, Ref. 200-334-9 Sigma). The control rats (EE + veh) received the same volume of saline solution containing 5% sucrose and 1% Tween 80 (sham treatment). Both Vandetanib and sham treatment were administered orally with a 1 ml syringe after an initial training phase with sucrose solution from P40 to P48. The animal’s body weight was recorded every 2nd day from P40 to P59, and additionally at P49, 1 day after changing the housing conditions.

### Behavioral Test

From P40, rats were handled daily by the investigator and trained to drink water from a 1 ml syringe for the later oral application of sucrose or Vandetanib.

Spatial and visual learning was assessed in all animals by an object displacement test (ODT) and an object replacement test (ORT) (Griffin et al., 2009) (Figure 1B). Rats were tested in a behavior-testing arena that consisted of a black circular open field (diameter: 1 m; height: 0.90 m) placed in a dimly lit room. Animals that were transported to JFJ were placed in their experimental housing conditions upon arrival and the following 2 days (P48–P50) were reserved for acclimatization. Starting from P50, rats were habituated daily to the empty testing arena, with 20 min of exploration in pairs for three consecutive days. Spatial memory testing (ODT) was performed at P54 and P55 and visual memory testing (ORT) at P57 and P58.

For the spatial test, two visual cues were fixed on the arena walls in quadrant positions north and south. For both tests, three objects (A, B, C) were constructed from toy bricks (Dreamstime) and fixed to the arena floor about 15 cm apart from the walls in three different quadrants. Objects were cleaned thoroughly between each trial to ensure the absence of olfactory cues. During the training phase, each animal was located in the middle of the arena facing the north quadrant and allowed to explore the objects (A, B, and C) for 3 × 5 min. The inter-trial resting phase was 5 min. One day after the training phase, one object (Object C) was moved to the empty quadrant (ODT) or replaced by another object (ORT) and a single trial was performed (5 min) to assess spatial memory retention of the object location or visual recognition of the novel object (Figure 1B). Exploration criteria were based on active exploration of the objects. For a positive count in this task the rats had to either touch the objects with their nose, whiskers or paws. The time spent to explore each different object was measured and the total exploration time calculated accordingly. Animals that explored less than 10 s during the training phase were not included into the test evaluation. The exploration times for the displaced (TD), new (TN), and familiar (TF) objects were measured and the different object discrimination indices (ODI_TD_/ODT_TN_) were calculated by the following two equations:

$$\begin{array}{c} {\mathrm{ODI}}_{\mathrm{TD}}\;=\;(\mathrm{TD}\;-\;({\mathrm{TF}}_{1}\;+\;{\mathrm{TF}}_{2})/2)/(\mathrm{TD}\;+\;({\mathrm{TF}}_{1}\;+\;{\mathrm{TF}}_{2})/2);\\ {\mathrm{ODI}}_{\mathrm{TN}}\;=\;(\mathrm{TN}\;-\;({\mathrm{TF}}_{1}\;+\;{\mathrm{TF}}_{2})/2)/(\mathrm{TN}\;+\;({\mathrm{TF}}_{1}\;+\;{\mathrm{TF}}_{2})/2). \end{array}$$

### Blood Samples and Hematocrit (Hct) Measurements

Blood samples were taken from anesthetized P59 rats by cardiac puncture with a 30G needle attached to a 1 ml heparinized syringe and the blood was immediately transferred into an Eppendorf tube (1 ml) prior to the transcardial whole-animal perfusion process.

The Hct was measured by use of blood sample-filled heparinized micro capillaries (Micro hematocrit tube 100, Assistant), which were immediately centrifuged for 5 min at 10 × 10^3^ rpm (Hematokrit 20 centrifuge, Hettich).

### Tissue Preparation for Immunohistochemistry

Rats were anesthetized by intraperitoneal injection of a lethal dose of sodium pentobarbital (150 mg/kg) and transcardially perfused with ice-cold phosphate-buffered saline solution (PBS) followed by fixation with 4% paraformaldehyde (PFA) in 0.1 M PBS. After perfusion, the brains were dissected and post fixed in 4% PFA overnight at 4°C. Thereafter the brains were stored in 30% sucrose in 0.1 M PBS. Serial 50 μm thick coronal sections (Figure 1C) were cut with a sliding microtome (Leica, Weltzar, Germany) and stored at -20°C in antifreeze solution.

### Butyryl Cholinesterase Histochemistry

To visualize and quantify neovascularization, brain tissue sections were histochemically processed with butyryl cholinesterase. Sections were washed twice in 0.1 M Tris-maleate buffer (TMB) (pH 6), acetylcholinesterase was inhibited for 20 min in 0.05 M 1,5-bis(4-allyldimethyl-ammoniumphenyl)-pentan-3-one dibromide (BW284CS1) (Ref: A-9013, Sigma-Aldrich, Spain), and sections were incubated overnight in the following incubation solution: butyryl thiocholine iodide (Ref: 108150250, Acros Organics, Barcelona, Spain) 1 mg/ml, 5% sodium citrate 0.1 M, 10% copper sulfate 30 mM, 10% BW284CS1 0.05 mM, 10% potassium ferricyanide 5 mM and 65% TMB 0.1 M. The next day, sections were washed in TMB, mounted on gelatine-coated slides, dehydrated and covered.

### Immunohistochemistry

Immunofluorescence stainings were performed to visualize neurogenesis [rabbit anti-Ki67 proliferation marker, ab15580, Abcam, 1:200 and goat anti-doublecortin X (DCX), SC-8066, Santa Cruz, 1:500], neurons (mouse anti-NeuN, MAB377, Chemicon, 1:1000) and apoptosis (rabbit anti-activated-caspase3, 9661, Cell Signaling Technology, 1:500). Sections were rinsed in 0.1 M PBS (H 7.4) and then incubated for 1 h in blocking solution containing 5% normal serum. Sections were then incubated overnight at 4°C with the corresponding primary antibody in 0.1 M PBS containing 0.3% Triton X-100 and 3% normal serum. The following day, sections were washed in 0.1 M PBS three times for 10 min and incubated for 30 min with the corresponding fluorochrome-conjugated secondary antibodies (donkey anti-rabbit Alexa Fluor 568, donkey anti-goat Alexa Fluor 488, goat anti-mouse Alexa Fluor 568, and goat anti-rabbit Alexa 488, Invitrogen) diluted 1:400 in 0.1 M PBS containing 0.3% Triton X-100 and 3% normal serum. DAPI (Ref. 28718-90-3, Merck) was added to counterstain the nuclei for 3 min before washing the secondary antibody 3 times for 10 min. Sections were mounted on gelatin-coated slides and cover-slipped with fluorescent mounting medium (Dako, Ref.S3023).

For stereological quantification, Ki67 and DCX were visualized by immunoperoxidase staining. Biotinylated secondary antibodies (donkey anti-rabbit IgG (H+L), and donkey anti-goat IgG (H+L)) diluted 1:300 in 0.1 M PBS containing 0.3% Triton X-100 and 3% normal serum were used. After incubation with the secondary antibody, slices were washed three times for 10 min in PBS. Sections were incubated with avidin-peroxidase-complex solution (Vector Labs) for 30 min at room temperature and washed again three times for 10 min in PBS. Sections were pre-incubated in DAB solution (50 mM Tris, 150 mM NaCl, 0.05% Triton X-100, 0.5 g/L DAB, pH 7.7) for 5 min under agitation, and DAB solution containing 0.01% H_2_O_2_ was added to sections. The reaction was stopped by washing in ice-cold PBS several times. Sections were mounted on gelatinized slides and dried overnight. After dehydration by immersion in increasingly concentrated ethanol solutions and clearing in xylene, slides were covered with Eukitt (Merck).

### Stereology

Neovasculature, Ki67+, and DCX+ cell densities were measured using the optical fractionator method with the help of the Mercator Image Analysis system (Explora Nova, La Rochelle, France). For vascularization, vessel length per unit volume was estimated by counting the number of intersections per unit volume, with the use of space balls (spherical) dissectors with a ratio of 25 μm separated by 120 μm. Ki67+ and DCX+ cell quantification was done in the granular cell layer of the dentate gyrus using grids of 20 μm × 20 μm separated by 40 μm (Figure 1C, left panel). Overall, 10 histological sections per animal were used. Total cell number was calculated with the following formula according to (West, 2012):

$$\begin{array}{c} N\;=\;\Sigma Q\;\times \;1/ssf\;\times \;1/asf\;\times \;1/hsf. \end{array}$$

*Q* = actual number of counted cells in a specimen, *N* = total estimate number, ssf = section sampling factor of 1/10, asf = area sampling factor, hsf = height of the sampling fraction.

### Image Acquisition

Whole-brain images from DAPI-labeled brains (Figure 1C) were taken using the Tiles tool from images acquired at 10×/0.45 NA in an epifluorescence microscope equipped with Apotome technology (Axio *Imager Z1*, Zeiss). *z*-stack images (optical sections 1 μm step size, total thickness of 16 mm) from DCX+/Ki67+ and caspase3+/NeuN+ immunofluorescence stainings were acquired with a confocal laser scanning microscope (LSM 700, Zeiss) using a 40×/1.4 NA objective and a pixel size of 112 nm × 112 nm. Four brain sections were imaged per rat and maximum intensity projections were created from *z*-stacks. Cell densities of caspase 3+/NeuN+ cells were directly quantified from each field of view. All imaging parameters were kept constant between groups. Images were processed with Fiji Image J Software (National Institutes of Health, United States).

### Statistical Analysis

Statistical data analysis was performed using SPSS statistical software (version 24.0, IBM). Prior to data analysis, the data sets were tested for normal distribution (Kolmogorov–Smirnov test) and homogeneity of variances (Levene’s test). Afterward, one-way ANOVA or two-way ANOVA with Bonferroni *post hoc* correction was applied. Data are presented as mean ± SD. Graphs were presented as two separate clusters. We determined on one hand the two independent variables normoxia and hypoxia over the dependent variable housing condition (SC and EE). On the other hand we represented the interaction between the two independent variables normoxia and hypoxia over the dependent variable drug application only in rats housed in enriched environment (EE + Veh and EE + VEGF inhibitor). Significance was declared at *P*-value < 0.05: ^∗^*P* < 0.05, ^∗∗^*P* < 0.01, ^∗∗∗^*P* < 0.001, ^∗∗∗∗^*P* < 0.0001.

## Results

### High Altitude Exposure in Juvenile Rats Increases Brain Microvasculature but Impairs Cognition and Causes Neuronal Apoptosis

**FIGURE 2:**
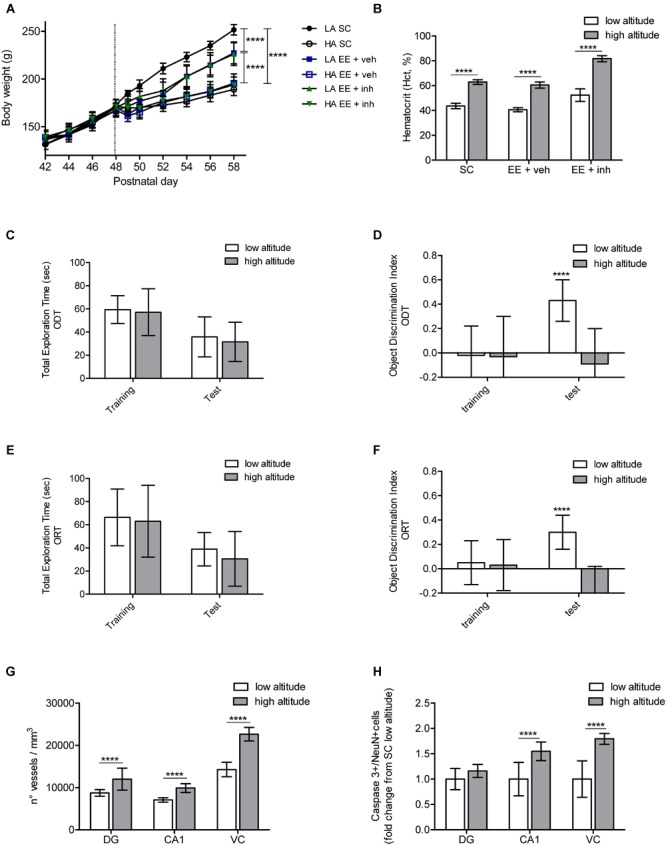
Physiological, cognitive, microvascular and neuronal survival changes caused by exposure to high altitude in Long Evans rats. **(A)** Body weight monitored every 2nd day between postnatal days 42 and 58 in rats exposed to low altitude (LA) or high altitude (HA), under SC and in enriched environment receiving either sucrose (EE + veh) or the inhibitor of VEGF signaling (EE + inh). **(B)** Hematocrit (Hct) values (in %), measured at the end of the experiment in rats exposed to low or high altitude kept either in SC, enriched environment receiving sucrose (EE + veh) or enriched environment receiving the inhibitor of VEGF signaling (EE + inh). **(C,D)** Changes in spatial memory tested by the Object Displacement Test (ODT) in rats exposed to low and high altitude kept in standard conditions. Shown is total exploration time **(C)** and object displacement discrimination index **(D)** during training and test. **(E,F)** Changes in visual memory tested by the Object Replacement Test (ORT) in rats exposed to low and high altitude kept in standard conditions. Shown is total exploration time **(E)** and object replacement discrimination index **(F)** during training and test. **(G)** Changes in microvasculature in brain DG, cornu ammonis (CA1) and visual cortex (VC) of rats exposed to low and high altitude kept in standard conditions. **(H)** Changes in neuronal apoptosis in brain DG, cornu ammonis (CA1) and visual cortex (VC) of rats exposed to low and high altitude kept in standard conditions. Statistics **(A,C–F)**: 2-way ANOVA and **(B,G,H)**: 1-way ANOVA (^∗∗∗∗^*P* < 0.0001) with *n* = 12; error bars indicate standard deviation (SD).

Physiological, cognitive and brain vascular and neuronal changes caused by high-altitude exposure in Long Evans juvenile rats was assessed (Figure 2).

As changes in body weight and hematocrit (Htc) are known to be influenced by high-altitude exposure, we first evaluated the impact of high altitude on these two factors. All groups of rats had similar body-weight gain at low altitude from P40 to P48. After fast ascent to high altitude all rats, independent of their housing condition, had a slower body-weight gain than rats at low altitude {2-way ANOVA [*F*_5,72_ (131.5), ^∗∗∗∗^*P* < 0.0001], Figure 2A}. Food consumption was measured by taking the food initial weight at P48, and then at P50 and P59. All rats at high altitude continued to eat less than low-altitude rats, resulting in a slower weight gain. No difference in body-weight gain was observed between housing conditions at high altitude. However, in the low-altitude groups, although food intake was similar throughout all the groups, rats kept in EE showed a slower weight gain than rats kept in SC, reaching statistical significance from day P52 to P59 {2-way ANOVA [*F*_2,72_ (58.47), ^∗∗∗∗^*P* < 0.0001], Figure 2A}. High-altitude exposure significantly increased the Hct from 43.6 ± 2.4 to 62.9 ± 1.9 in rats kept in SC, from 40.6 ± 1.6 to 60.5 ± 2.5 in rats kept in EE, and from 52.4 ± 5.1 to extremely high levels of 81.7 ± 2.4 in rats kept in EE receiving VEGF inhibitor (EE + inh) (ANOVA, ^∗∗∗∗^*P* < 0.0001, Figure 2B). In rats kept in EE receiving VEGF inhibitor (EE + inh), Hct increased significantly even in low-altitude reared rats {2-way ANOVA [*F*_1,44_ (319), ^∗∗∗∗^*P* < 0.0001], not shown}.

We assessed the impact of high altitude on spatial hippocampus-dependent memory and visual memory using an object-displacement test (ODT) and object-replacement test (ORT), respectively, in SC-housed rats. High altitude did not affect the total exploration time of rats during ODT training and test (Figure 2C). During training all rats explored the three objects equally (not shown), but failed to recognize the displacement of the object {2-way ANOVA [*F*_1,24_ (12.06), ^∗∗∗^*P* = 0.001], Figure 2D}. Similarly, the total exploration time of rats during ORT training and test did not differ between low and high altitude (Figure 2E). Rats spent the same time exploring all three objects during training (not shown), but rats at high altitude failed to recognize the object replacement {2-way ANOVA [*F*_1,24_ (22.77), ^∗∗∗∗^*P* < 0.001], Figure 2F}.

A significant increase in vessel density in the dentate gyrus, the CA1 area of the hippocampus, and in the visual cortex was observed in SC rats exposed to high altitude (ANOVA, ^∗∗∗∗^*P* < 0.0001, Figures 2G, 4A,D,G left panels). Thus it appears that the cognitive impairment caused by high altitude in rats was not prevented by enhanced vascular density.

Finally, we evaluated the effect of high-altitude exposure on neuronal apoptosis in the CA1 area of the hippocampus and the visual cortex in SC-housed rats. Neuronal apoptosis was enhanced in both areas (ANOVA, ^∗∗∗∗^*P* < 0.0001, Figures 2H, 6A). This suggests that neuronal apoptosis is likely to account for the cognitive impairment, with CA1 hippocampal apoptosis affecting mainly spatial memory, and visual cortex apoptosis affecting visual memory.

### Enriched Environment Including Voluntary Exercise (EE) During High-Altitude Exposure Prevents Impairment of Spatial and Visual Memory via VEGF Signaling

**FIGURE 3:**
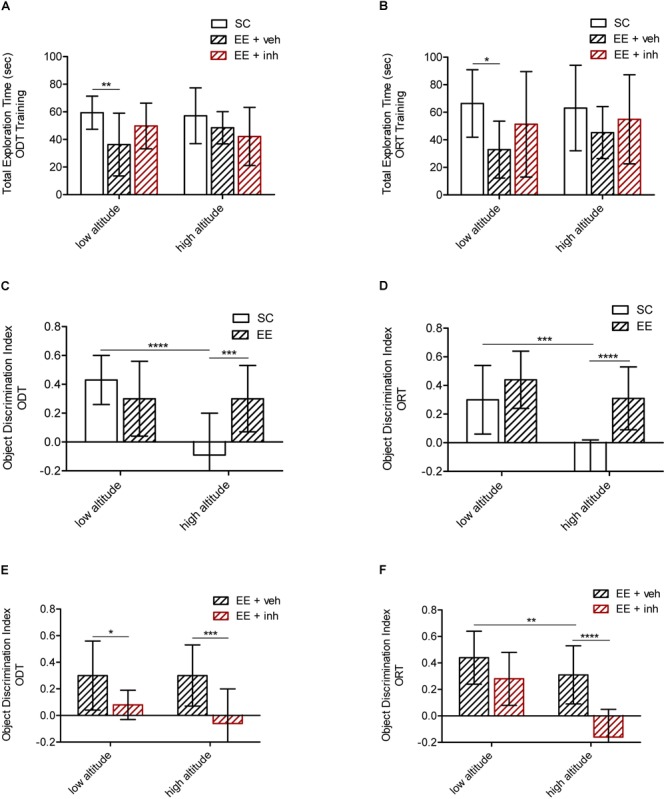
Effect of enriched environment (EE) and the inhibitor of VEGF signaling on spatial and visual memory in high-altitude exposed rats. **(A,C,E)** Spatial memory tested by the ODT. Shown is total exploration time during training in low altitude and high altitude exposed rats **(A)** as well as the object displacement discrimination index during test **(C,E)** in rats housed in standard conditions (SC, white bar) or in enriched environment (EE, patterned bar) **(C)**, as well as in rats in enriched environment receiving either sucrose (EE + veh, black patterned bar) or the inhibitor of VEGF signaling (EE + inh, red patterned bar) **(E)**. **(B,D,F)** Visual memory tested by the ORT. Shown is total exploration time during training in low altitude and high altitude exposed rats **(B)** as well as the object replacement discrimination index during test **(D,F)** in rats housed in standard conditions (SC, white bar) or in enriched environment (EE, patterned bar) **(D)**, and in rats housed in enriched environment receiving sucrose (EE + veh, patterned bar) or in enriched environment receiving the inhibitor of VEGF signaling (EE + inh, red patterned bar) **(F)**. Statistics 2-way ANOVA (^∗∗∗∗^*P* < 0.0001, ^∗∗∗^*P* < 0.001, ^∗∗^*P* < 0.01, ^∗^*P* < 0.05) with *n* = 12; error bars indicate SD.

We determined the impact of environmental enrichment including exercise (EE) in recovering spatial hippocampus-dependent memory (ODT) and visual memory (ORT) in high-altitude exposed rats (Figure 3).

Rats reared in EE at low altitude explored generally less than SC animals during ODT training {2-way ANOVA [*F*_2,72_ (5.21), ^∗∗^*P* < 0.01], Figure 3A} as well as during ORT training {2-way ANOVA [*F*_2,72_ (4.51),^∗^*P* < 0.05], Figure 3B}. The total exploration time during test was equal in all animals groups (not shown). During training for both tests, all three objects were equally explored across the groups (not shown). During test, low altitude rats housed in SC and EE were able to recognize the displaced object (ODT) as well as the replaced object (ORT). Although the total exploration time was significantly shorter in EE rats, it was sufficient for correct object recognition during the test, reflecting the pro-plasticizing effect of EE. Rats kept in EE at high altitude were also able to recognize the displaced object (ODT) {2-way ANOVA [*F*_1,48_ (9.7), ^∗∗∗^*P* < 0.001], Figure 3C} as well as the replaced object (ORT) {2-way ANOVA [*F*_1,48_ (27.5), ^∗∗∗∗^*P* < 0.0001], Figure 3D}. Thus, EE appears to prevent the loss in cognition caused by high altitude.

We next evaluated the possible involvement of VEGF signaling in the EE-mediated recovery of memory after high-altitude exposure. EE-housed rats that received the inhibitor of VEGFR-2 (EE + inh) daily, spent the same time exploring during training and test as EE rats receiving sucrose (EE + veh) did (Figures 3A,B), but they failed to recognize the ODT at either altitude {2-way ANOVA [*F*_1,48_ (20.2), ^∗∗∗∗^*P* < 0.0001], Figure 3E}, and failed to recognize the ORT at high altitude {2-way ANOVA [*F*_1,48_ (28.48), ^∗∗∗∗^*P* < 0.0001], Figure 3F}. Thus, it appears that VEGF signaling is required to preserve spatial memory at low altitude and to prevent the impairment of spatial and visual memory caused by high altitude.

### Inhibition of VEGF Signaling Prevents the EE-Mediated Increase in Brain Microvasculature at Low and High Altitude

**FIGURE 4:**
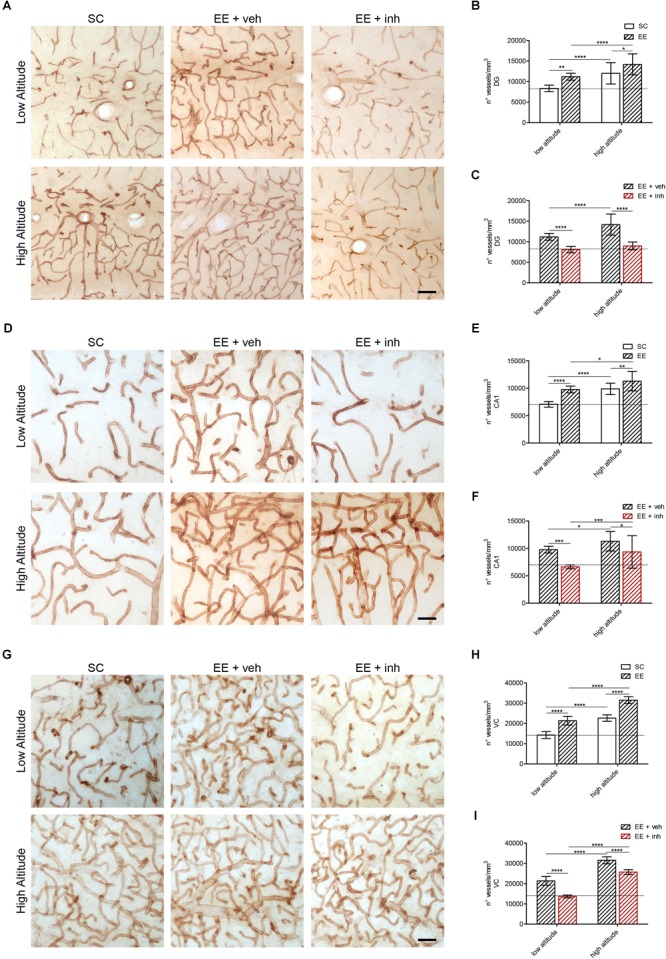
Microvascular changes caused by enriched environment, and the inhibitor of VEGF signaling in DG, cornu ammonis (CA1) hippocampus and VC of rats exposed to high altitude. **(A)** Representative images of vessels stained with butyrylcholinesterase (BChE) in the hippocampus DG area of rats exposed to low (upper panel) or high altitude (lower panel) under SC and in enriched environment receiving either sucrose (EE + veh) or the inhibitor of VEGF signaling (EE + inh). **(B,C)** Blood vessel densities in the DG area of low- and high-altitude exposed rats kept in standard conditions (SC, white bar) or in enriched environment (EE, patterned bar) **(B)** as well as in enriched environment receiving either sucrose (EE + veh, black patterned bar) or the inhibitor of VEGF signaling (EE + inh, red patterned bar) **(C)**. **(D)** Representative images of vessels stained with butyrylcholinesterase (BChE) in the hippocampus cornu ammonis (CA1) area of rats exposed to low (upper panel) or high altitude (lower panel) under SC and in enriched environment receiving either sucrose (EE + veh) or the inhibitor of VEGF signaling (EE + inh). **(E,F)** Blood vessel densities in the cornu ammonis (CA1) area of low- and high-altitude exposed rats kept in standard conditions (SC, white bar) or in enriched environment (EE, patterned bar) **(E)** as well as in enriched environment receiving either sucrose (EE + veh, black patterned bar) or the inhibitor of VEGF signaling (EE + inh, red patterned bar) **(F)**. **(G)** Representative images of vessels stained with BChE in the layer 4 VC of rats exposed to low (upper panel) or high altitude (lower panel) under SC and in enriched environment receiving either sucrose (EE + veh) or the inhibitor of VEGF signaling (EE + inh). **(H,I)** Blood vessel densities in the layer 4 VC of low and high altitude exposed rats kept in standard conditions (SC, white bar) or in enriched environment (EE, patterned bar) **(H)** as well as in enriched environment receiving either sucrose (EE + veh, black patterned bar) or the inhibitor of VEGF signaling (EE + inh, red patterned bar) **(I)**. Scale bar **(A,D,G)**: 50 mm. Statistics **(B,C,E,F,H,I)**: 2-way ANOVA (^∗∗∗∗^*P* < 0.0001, ^∗∗∗^*P* < 0.001, ^∗∗^*P* < 0.01, ^∗^*P* < 0.05) with *n* = 12; error bars indicate SD.

We assessed the effect of EE on vascularization in the dentate gyrus (Figures 4A–C), CA1 hippocampus (Figures 4D–F) and the visual cortex layer 4 microvasculature (Figures 4G–I) after ascent to high altitude, as well as the impact of inhibition of VEGF (Figures 4C,F,I).

As reported above, a significant increase in vessel density was observed in dentate gyrus, CA1 hippocampus and visual cortex of all rats exposed to high altitude (Figures 4A,B,D,E,G,H). Also rats kept in EE receiving sucrose (EE + veh) showed significantly higher blood vessel densities in dentate gyrus, CA1 hippocampus and visual cortex than SC rats at both low and high altitude (Figures 4A,B,D,E,H). 2-way ANOVA: [*F*_1,48_ (42.6), ^∗∗∗∗^*P* < 0.0001, Figure 4B], [*F*_1,48_ (43.7), ^∗∗∗∗^*P* < 0.0001, Figure 4E], and [*F*_1,48_ (742), ^∗∗∗∗^*P* < 0.0001, Figure 4H]. The effect of EE on increasing vessel densities was reduced by inhibition of VEGF signaling in all areas at both low and high altitudes (Figures 4A,C,D,F,G,I). 2-way ANOVA: [*F*_1,48_ (94.53), ^∗∗∗∗^*P* < 0.0001, Figure 4C], [*F*_1,48_ (24.98), ^∗∗∗∗^*P* < 0.0001, Figure 4F], and [*F*_1,48_ (229), ^∗∗∗∗^*P* < 0.0001, Figure 4I]. Therefore, the EE-mediated increase in microvasculature is VEGF dependent. By contrast, the increase in vessel densities caused by high altitude was only prevented by inhibition of VEGF signaling in the dentate gyrus (Figure 4C), indicating that proliferative (neurogenic) brain areas require VEGF signaling to increase vascular densities in hypoxia. However, in CA1 hippocampus and visual cortex, other oxygen-dependent factors might be involved.

### Inhibition of VEGF Signaling Prevents Dentate Gyrus Neurogenesis at High Altitude

**FIGURE 5:**
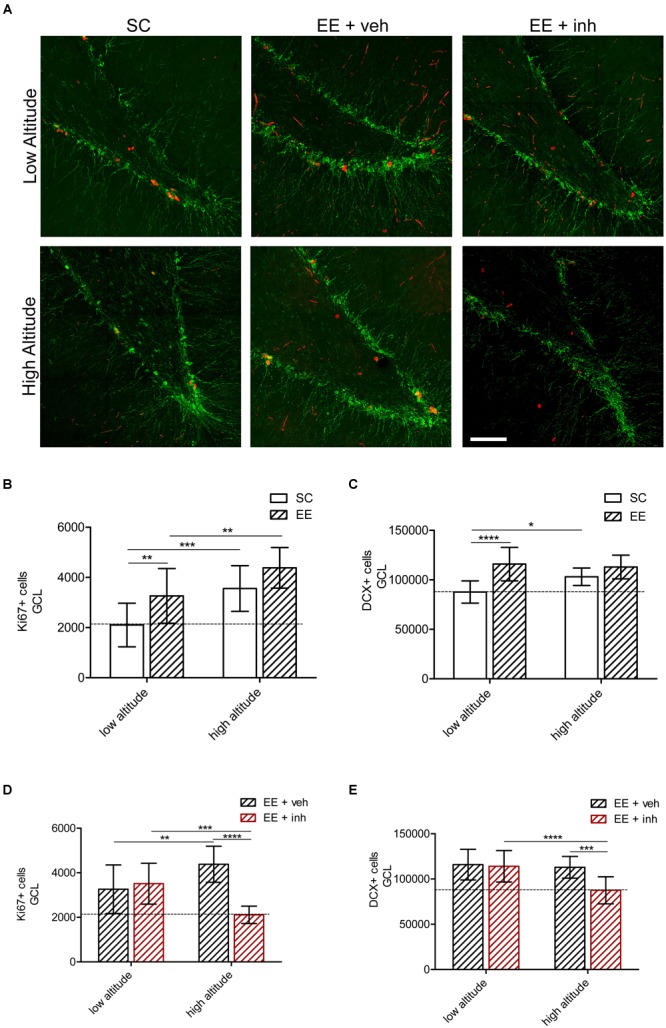
Effect of EE and VEGF signaling inhibitor in DG neurogenesis in rats exposed to high altitude. **(A)** Confocal representative images of proliferation (Ki67, red) and mitotic neurons (doublecortin, DCX, green) in rats exposed to low altitude (upper panel) or high altitude (lower panel) kept under SC or in enriched environment either receiving sucrose (EE + veh) or the inhibitor of VEGF signaling (EE + inh). **(B,C)** Quantification of Ki67+ cells in the granular cell layer (GCL) of rats exposed to low or high altitude under standard conditions (SC, white bar) or in enriched environment (EE, patterned bar) **(B)** as well as in enriched environment receiving either sucrose (EE + veh, black patterned bar) or the inhibitor of VEGF signaling (EE + inh, red patterned bar) **(C)**. **(D,E)** Quantification of doublecortin (DCX) positive cells of rats exposed to low or high altitude under standard conditions (SC, white bar) or in enriched environment (EE, patterned bar) **(D)** as well as in enriched environment receiving either sucrose (EE + veh, black patterned bar) or the inhibitor of VEGF signaling (EE + inh, red patterned bar) **(E)**. Scale bar **(A)**: 100 mm. Statistics **(B–E)**: 2-way ANOVA (^∗∗∗∗^*P* < 0.0001, ^∗∗∗^*P* < 0.001, ^∗∗^*P* < 0.01, ^∗^*P* < 0.05) with *n* = 12; error bars indicate SD.

Considering that VEGF signaling is required to maintain vascular densities in the dentate gyrus at high altitude, we evaluated the impact of high altitude and EE in neurogenesis, i.e., proliferating cells (Ki67), and neuronal differentiation (DCX) in the internal granular cell layer of the dentate gyrus. Representative images were taken from double-immunofluorescence labeling (Figure 5A) and quantification was done in sections processed for immunoperoxidase staining (Figures 5B–E).

Exposure to high altitude increased the number of proliferating (Ki67+) cells by 69% in SC-housed rats. Likewise, at both low and high altitude, EE-housed rats receiving sucrose (EE + veh) showed 55% (low altitude) and 100% (high altitude) more Ki67+ cells than SC rats {2-way ANOVA [*F*_1,48_ (13.86), ^∗∗∗^*P* < 0.001], Figure 4B}. High altitude also caused a 1.2-fold increase in DCX+ cells in SC rats. At both low and high altitude, EE-housed rats receiving sucrose (EE + veh) showed a 30% increase in DCX+ cells. High altitude and EE caused an equal increase in DCX+ cell numbers.

When the inhibitor of VEGF signaling was given to EE-housed rats at low-altitude, the number of Ki67+ cells remained 67% higher than SC rats. In high-altitude rats, however, the number was reduced, and equal to that of the low-altitude SC-housed rats {2-way ANOVA [*F*_1,48_ (17.24), ^∗∗∗^*P* < 0.001], Figure 5D}. Also, DCX+ cells were reduced only in high-altitude exposed rats, while rats at low altitude kept a 30% increase {2-way ANOVA [*F*_1,48_ (9.3), ^∗∗^*P* < 0.01], Figure 5E}. This indicates that EE-mediated VEGF signaling is required to maintain neurogenesis at high altitude.

### EE-Mediated VEGF Signaling Is Neuroprotective at High Altitude

**FIGURE 6:**
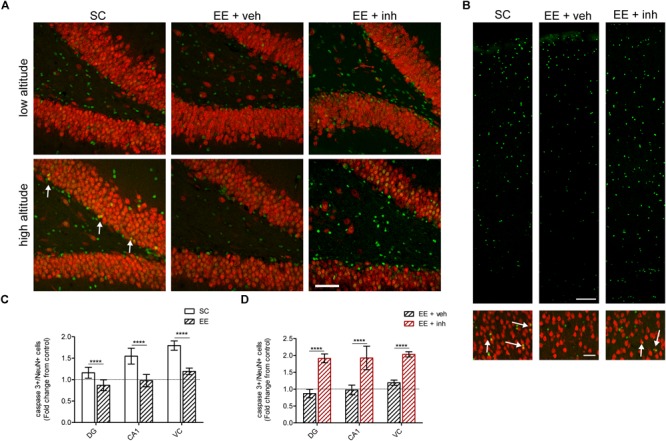
Effect of enriched environment and VEGF signaling inhibition in neuronal apoptosis in DG, cornu ammonis (CA1) of the hippocampus and VC in rats exposed to high altitude. **(A)** Confocal representative images of neurons (NeuN, red) and apoptotic cells (activated caspase 3, green) in the dentate gyrus of rats exposed to low and high altitude kept under SC or in enriched environment either receiving sucrose (EE + veh) or the inhibitor of VEGF signaling (EE + inh). **(B)** Confocal representative images of apoptotic cells (activated caspase 3, green) in the layers 1–6 of the VC (upper panel), and of apoptotic neurons [NeuN (red) colocalized to caspase 3 (green)] in the layer 4 of the VC (lower panel, arrows) of rats exposed to high altitude kept under SC or in enriched environment either receiving sucrose (EE + veh) or the inhibitor of VEGF signaling (EE + inh). **(C)** Quantification of caspase 3+ apoptotic cells in DG, cornu ammonis (CA1) and VC of rats exposed to high altitude under standard conditions (SC, white bar) and in enriched environment (EE, patterned bar). **(D)** Quantification of caspase 3+ apoptotic cells in DG, cornu ammonis (CA1) and VC of rats exposed to high altitude under enriched environment receiving either sucrose (EE + veh, black patterned bar) or the inhibitor of VEGF signaling (EE + inh, red patterned bar). Scale bar **(A,B)**: 100 mm. Statistics **(C,D)**: 2-way ANOVA (^∗∗∗∗^*P* < 0.0001) with *n* = 12; error bars indicate SD.

Next we analyzed the neuroprotective effect of EE at high altitude as well as the impact of inhibition of VEGF signaling. Neuronal apoptosis was assessed by immunofluorescence analysis of activated caspase 3 expression in neurons (NeuN+) in the dentate gyrus (Figure 6A) and the visual cortex (Figure 6B).

As shown above, high-altitude exposure increased neuronal apoptosis in rats housed in SC. In contrast, EE reduced the number of apoptotic neurons at high altitude (ANOVA, ^∗∗∗∗^*P* < 0.0001, Figure 6C). In EE-housed rats at high-altitude the number of CA1 hippocampus and visual cortex NeuN+ neurons was 18% higher compared to SC-housed rats {2-way ANOVA [*F*_1,48_ (24.41), ^∗∗∗∗^*P* < 0.0001], CA1 hippocampus, and [*F*_1,48_ (26.64), ^∗∗^*P* < 0.01], visual cortex}. This indicates that EE is neuroprotective and prevents neuronal loss caused by high altitude.

When the inhibitor of VEGF signaling was given to EE-housed rats, neuronal apoptosis was significantly increased in high-altitude exposed rats {2-way ANOVA [*F*_1,48_ (61.99), ^∗∗∗∗^*P* < 0.0001], Figure 6D}, causing a significant reduction in dentate gyrus granule cells and NeuN+ cells in CA1 hippocampus and visual cortex, to values similar to SC {2-way ANOVA [*F*_1,48_ (45.7), ^∗∗∗∗^*P* < 0.0001]}. Taken together, our results clearly showed that VEGF signaling is required to mediate the neuroprotective effects of EE against neuronal loss caused by exposure to high altitude.

## Discussion

We evaluated the impact of EE and VEGF signaling on spatial and visual memory, as well as on neovascularization, neurogenesis, and neuronal apoptosis after prolonged exposure to high altitude. In order to address this, we have interfered with VEGF signaling by blocking its receptor pharmacologically with Vandetanib. The main findings of our study are summarized in Table 1. Briefly, VEGF signaling is required for the adaptation of the brain to high altitude by reducing neuronal apoptosis, maintaining the neurogenic pool and preserving cognition.

**Table 1:** Summary of brain changes caused by high altitude exposure, enriched environment and inhibition of VEGF.

Measured parameters (compared to low altitude SC)	Low altitude		High altitude		
	
	EE	EE + inh	SC	EE	EE + inh
Neovasculature/angiogenesis	+	–	+	++	+ -(DG)
Neurogenesis	+	+	+	++	–
Spatial memory	Yes	No	No	Yes	No
Visual memory	Yes	Yes	No	Yes	No
Neuronal apoptosis (activated caspase 3)	–	–	+	–	+

High-altitude and EE both stimulate neurogenesis and angiogenesis. Yet, in high-altitude, the increase in neurogenesis and angiogenesis is not a causal link to memory improvement. We instead postulate that a secondary effect on neuronal networks might be implicated in the cognitive improvement and that the preservation of cognition at high-altitude is due, at least in part, to the pro-survival effect of EE-mediated VEGF. We also showed that neurogenesis, angiogenesis and cognition are differently affected by inhibition of VEGF signaling in EE-housed rats at low and high altitude. At low altitude inhibition of VEGF signaling impairs spatial memory and angiogenesis without affecting neurogenesis. Conversely, at high altitude inhibition of VEGF signaling impairs spatial and visual memories as well as angiogenesis and neurogenesis. In the CA1 hippocampus and visual cortex, high altitude-induced angiogenesis is only partially reduced by inhibition of VEGF signaling, suggesting the involvement of other hypoxia-inducible factors. Inhibition of VEGF signaling also prevented the decrease in apoptosis caused by EE, and caused impairment in spatial and visual memory. Therefore, we were able to show a positive impact of EE-mediated VEGF signaling in preventing high altitude-induced memory impairment and neuronal loss, and discovered a pivotal role of VEGF signaling in the maintenance of neurogenesis after high-altitude exposure.

### Effect of High Altitude, EE and Inhibition of VEGF Signaling on Body Weight Gain and Hematocrit

We evaluated the longitudinal impact of prolonged high altitude exposure on body weight gain and on hematocrit levels. Rats exposed to high altitude, independent of their housing conditions, showed reduced weight gain, which was primarily attributed to the reduced food intake potentially caused by hypoxia-induced anorexia (Py et al., 2005). The loss in body weight could reflect impairment in normal development; however, cognitive impairment was not causally related.

Hypoxia and inhibition of VEGF signaling both led to an increase in hematocrit, probably due to loss of plasma volume (not measured) and an increase in red blood cells (stress erythropoiesis). It is well known that exposure to chronic continuous hypoxia causes an increase in hematocrit to ensure a higher systemic vascular conductance of oxygen (O_2_) (Martinez-Bello et al., 2011). The effect of VEGF signaling inhibition on hematocrit has already been reported in human trials, where an increase in red blood cells and erythropoietin production occurred upon antiangiogenic treatments with VEGF inhibitors (Bhatta et al., 2013).

### Effect of High Altitude, EE and Inhibition of VEGF Signaling on Angiogenesis and Neurogenesis

The vascular network of the body is in charge of maintaining metabolic homeostasis by supplying oxygen and nutrients. Angiogenesis is one of the key mechanisms of high-altitude adaptation (Gassmann et al., 2016), and it is believed that cognitive function can be preserved by this mechanism (Ding et al., 2006). We found a significant neovascularization in all analyzed brain areas of high-altitude exposed rats. We further suggest that the dentate gyrus is a brain area in which VEGF signaling is of particular importance for hypoxia-induced angiogenesis. The inhibition of VEGF signaling completely prevented changes in vessel density in the dentate gyrus. Indeed, stem cells and neural progenitor cells in the dentate gyrus are VEGF secretory cells and may therefore locally stimulate angiogenesis and neovascularization (Kirby et al., 2015). In the visual cortex and CA1 hippocampus, inhibition of VEGF signaling only partially blocked neovascularization caused by hypoxia, suggesting the involvement of other hypoxia-inducible factors such as erythropoietin (Ribatti et al., 1999). EE-induced angiogenesis in high-altitude housed rats and inhibition of VEGF signaling blocked the EE-mediated angiogenic action in all evaluated brain areas, indicating that EE-mediated angiogenesis is exclusively VEGF-mediated.

Angiogenesis and neurogenesis are closely related processes, since neural stem cells are able to secrete VEGF, which allow these cells to regulate neurogenesis in their own niche (Kirby et al., 2015). In our setting, both high altitude and EE increased neurogenesis. However, inhibition of VEGF signaling blocked the neurogenic action only in high-altitude exposed rats, suggesting that the auto regulatory effect of VEGF was lost, and a paracrine signaling of VEGF is required for the maintenance of the neurogenic pool at high altitude. Indeed, high levels of VEGF are found mainly in astrocytes upon hypoxic exposure (Ortuzar et al., 2013), indicating that astrocyte-derived VEGF signaling potentially regulates neurogenesis. The current results suggest a different dependency of the neurogenic niche on VEGF signaling at low and high altitude.

Although a positive correlation between neurogenesis and cognition was previously demonstrated (Ding et al., 2006), the effect of VEGF on neurogenesis and memory are not causally related. For example, VEGF-induced angiogenesis *per se* does not necessarily predict an increase in neurogenesis, and inhibition of VEGF signaling has been shown to impair memory without affecting neurogenesis (Licht et al., 2011). Additionally, the effect of VEGF overexpression or blockade on memory gain/loss can be measured a couple of days after the initial induction/blockade, a time window considered to be too short to account for neurogenesis being the main responsible factor (Foscarin et al., 2011, 2012).

The possibility of activating hippocampal progenitor cells by hypoxia and subsequently boosting neurogenesis in the adult brain is being postulated as an exciting therapeutic strategy to ameliorate cognitive impairment in neurodegenerative diseases like Alzheimer’s, Parkinson’s and ischemic stroke (Han et al., 2016). Importantly, our results suggest that hypoxia-induced neurogenesis alone is not sufficient to improve cognition. But when combined with enriched environment and exercise, it is conducive to preventing neuronal apoptosis and stimulating brain network activity. Further evidence that neurogenesis is not the main factor responsible for cognitive improvement is provided by our low-altitude results, where blocking VEGF did not affect neurogenesis but impaired spatial memory. Our findings indicate that VEGF signaling in the dentate gyrus of the adult brain is crucial for the adaptation to high altitude and to preserve hippocampus-related memory at low altitude. These results highlight the risk of VEGF inhibitors usage in cancer therapy by providing evidence that inhibition of VEGF signaling impairs hypoxia-induced neurogenesis and angiogenesis as well as causing cognitive impairment. The dysfunction in VEGF signaling caused by VEGF inhibitors, and its potential adverse consequences on cognition, should therefore be taken into consideration when patients receive this kind of treatment, and ascent to high altitude should probably not be recommended, at least not during the VEGF inhibition treatment phase.

### Effect of High Altitude, EE and Inhibition of VEGF Signaling on Neuroprotection and Cognition

High-altitude exposure caused an increase in neuronal apoptosis, which was correlated with a reduction in neurons in the CA1 hippocampus and visual cortex in SC rats. EE reduced neuronal apoptosis, and inhibition of VEGF signaling prevented the anti-apoptotic effect of EE conditions, which indicates a pivotal role of VEGF signaling in EE-induced neuroprotection. It was previously shown that VEGF exerts neuroprotective actions directly through VEGFR-2 receptors expressed on neurons subjected to hypoxia, activating the phosphatidylinositol 3-kinase (PI3K)/Akt signal transduction system, which leads to the inhibition of proapoptotic signaling effectors such as Bad, caspase-9 and caspase-3 (Gora-Kupilas and Josko, 2005). We observed an increase in neuronal cells in the dentate gyrus, CA1 hippocampus and visual cortex under EE, suggesting that increased neuronal activity under EE and exercise induces cerebral VEGF expression, which exerts an acute effect on survival of neurons and on angiogenesis and neurogenesis. Furthermore, we could show that blocking VEGFR-2 led to a decreased learning capacity. Thus, the detrimental effect of high altitude in cognition is attributed, at least in part, to neuronal apoptosis which can be prevented by EE and exercise. Exercise in humans is also a potential therapeutic strategy to counteract cognitive impairment. Indeed, cognitive performance improves during exercise under both normoxic and hypoxic conditions (Komiyama et al., 2017). Thus, similarly to cerebrally expressed erythropoietin (Schuler et al., 2012), also VEGF might counteract the reduced cognition capacity at high altitude, allowing better chances of survival in hypoxic conditions.

The hippocampus is one of the most hypoxia-sensitive areas of the brain and it is a crucial region for spatial memory formation (Kandel, 2001). The cause of hippocampal memory impairment at high altitude is less well understood but, as previously mentioned, the integrity of brain function depends on a continuous and sufficient oxygen supply (Komiyama et al., 2017), and therefore the brain vascular network plays an essential role in balancing oxygen availability versus energy demands in hypoxic stress conditions. Indeed, EE prevented the loss of spatial and visual memory caused by exposure to high altitude in rats. By contrast, VEGF inhibition in EE-housed rats caused spatial memory impairment, indicating that VEGF is important in preserving hippocampal-dependent memory. Importantly, it has been shown that VEGF promotes memory and increases synaptic strength independently of its effects on neurogenesis and angiogenesis (Licht et al., 2011). Thus, enrichment-induced improvement of hippocampal-mediated memory has been related, among other effects, to increased synaptic plasticity (Kempermann et al., 1997). The positive effect of EE on survival and synaptic strength could also be attributed to the observed improvement in visual memory.

## Conclusion

We conclude that the exposure to an enriched environment including exercise is advantageous to the recovery of impaired cognitive function at high altitude. We further propose that VEGF signaling is crucial to the preservation of neurons and neovascularization, and also to maintaining neurogenesis at high altitude. Overall, our findings extend the knowledge of the complex response of the neurovascular unit to high-altitude stress and furthermore show the importance of VEGF signaling for angiogenesis, neurogenesis, neuroprotection, and spatio-visual memory in hypoxic conditions.

## Author’s Note

This article is dedicated to Enrike Gutierrez Argandoña (1966–2014), who started this work.

## Author Contributions

CK-H and ESG performed the morphological analysis. HB performed the stereological analysis. DK, MT, TH, and ESG performed the behavioral tests and the transport of the animals to Jungfraujoch. MT, TH, MG, and ESG discussed the results and analysis. ESG interpreted the findings and supervised this work. All authors discussed the results and contributed to the final manuscript.

## Conflict of Interest Statement

The authors declare that the research was conducted in the absence of any commercial or financial relationships that could be construed as a potential conflict of interest.
